# Editorial: Best practice approaches in women's sports

**DOI:** 10.3389/fpsyg.2023.1260044

**Published:** 2023-10-31

**Authors:** Rubén Maneiro, Mario Amatria, Vasilis Armatas, Claudio A. Casal, José Luís Losada, Antonio Ardá

**Affiliations:** ^1^Department of Science of Physical Activity and Sport, Pontifical University of Salamanca, Salamanca, Spain; ^2^School of Physical Education and Sport Science, National and Kapodistrian University of Athens, Athens, Greece; ^3^Department of Physical Activity and Sport Science, Catholic University of Valencia San Vicente Mártir, Valencia, Spain; ^4^Department of Social Psychology and Quantitative Psychology, University of Barcelona, Barcelona, Spain; ^5^Department of Physical and Sports Education, University of La Coruña, La Coruña, Spain

**Keywords:** women's sport, performance analysis, physical exercise, training, competition

## Introduction

The first scientific articles on women's sport began to be published in the 1980s and their object of research focused on thematic areas such as the physiology and anthropometric values of female players (Iván-Baragaño and Maneiro, 2023).

The measurement of parameters such as height, body fat percentage, maximal oxygen consumption or lactate concentration were the leitmotivs during the 1990s and early 2000s (Kirkendall, 2007). In the last decade of the last century, the number of publications increased slightly, although the total number remained relatively small compared with that of the corresponding publications dealing with men's sport.

According to the PUBMED database, a significant and exponential increase in studies of women's sport has been observed in recent years. More specifically, it can be stated that a larger number of studies have been published in the last decade (2013–2023, *n* = 96,882) than in the entire period since the end of the Second World War (1945–2012, *n* = 81,588).

The present subject of research entitled “*Best practice approaches in women's sports*” was conceived with the aim of providing a forum through which scientists can submit their research studies of women's sport in terms of all its aspects and the full range of categories and types of research. As a result, it can be noted that the topic has had a significant impact, given that eight articles by 52 different authors have been published, with a total number of more than 19,000 visits to the related website and more than 3,000 downloads. This has made it possible to expand and develop the range of information available with regard to both individual sports (four studies presented) and collective sports (four scientific papers presented).

## Overview of contributions

The analysis of sporting performance is the subject on which most attention was focused in the studies concerned. This is no coincidence, given the lack of adequately researched scientific literature concerning women's sport in any of its forms and disciplines. In particular, there was a balance among the proposals submitted, since four works dealt with individual sports: jiu-jitsu (Santos et al.); judo (Barreto et al.); pentathlon (Qiao et al.); and speed skating (Liu et al.). And, on the other hand, another four have addressed collective sports: football (Iván-Baragaño et al.; Zhang et al.; Costa et al.) and volleyball (Slovák et al.).

With regard to martial arts, the paper entitled “*The effects of weight categories on the time-motion analysis of female high-level judo athletes between the 2016 and 2020 Olympic cycles*” analyzed 1,332 high-level judo bouts, concluding that the temporal behavior of the contest changed in nature between Olympic cycles. The study entitled “*Effects of weight divisions in time-motion of female high-level Brazilian Jiu-jitsu combat behaviors*” was also particularly relevant. This study compared continuous high-level female Brazilian Jiu-jitsu maneuvers in terms of time and frequency, taking into consideration weight categories. The main findings showed that the Super Heavyweight category was characterized by a shorter grip time than other weight categories. By contrast, the Rooster category was characterized by longer grip, transition and attack times and frequencies than the Light, Middle and Heavyweight categories.

Another interesting paper focusing on individual sports is entitled “*The effects of 8-week complex training on lower-limb strength and power of Chinese elite female modern pentathlon athletes*”. The study aimed to analyse the effects of complex training (CT) on lower limb strength in female modern pentathlon athletes. The study concluded that CT in combination with regular training (RT) gave rise to an improvement in lower-limb strength and power in elite female modern pentathlon athletes.

The latest study focusing on individual sports is entitled “*Construction of Women's All-Around Speed Skating Event Performance Prediction Model and Competition Strategy Analysis Based on Machine Learning Algorithms*”. The study aimed to explore the feasibility and effectiveness of machine learning algorithms for predicting women's full speed skating event performance, concluding that the ML algorithm was demonstrated to be a feasible method for predicting women's overall performance in speed skating competitions.

As far as collective sports are concerned, three studies focused on the analysis of football, but from different perspectives. On the one hand, the study entitled “*Future horizons in the analysis of technical-tactical performance in women's football: a mixed methods approach to the analysis of in-depth interviews with professional coaches and players*”, conceived with regard to the context of mixed methods, deals with indirect observation applied to interviews with high performance football players and coaches, with the aim of finding variables associated with success. Another work analyzing an aspect of football is “*The effect of the video assistant referee (VAR) on referees' decisions at FIFA Women's World Cups*”, which analyses how video refereeing affected refereeing decisions during the FIFA World Cups in 2015 and 2019. Finally, training in women's football was also a subject of analysis within this topic. Specifically, the paper entitled “*Training in women soccer players: a systematic review on training load monitoring*”, where they concluded that the training load (TL) during training sessions in women football players is very low, and it is currently very difficult to consider evidence-based practices.

Finally, the study entitled “*External focus of attention enhances arm velocities during volleyball spike in young female players*”, analyses the attentional focus during the spike in female volleyball players, finding that a significant benefit was found from the start of the wind-up phase to the acceleration phase (prior to ball-hitting) under external focus conditions.

## Author contributions

RM: Writing—original draft, Writing—review & editing. MA: Writing—review & editing. VA: Supervision, Conceptualization, Resources, Writing—review & editing. CC: Supervision, Writing—review & editing. JL: Supervision, Validation, Visualization, Writing—review & editing. AA: Conceptualization, Supervision, Writing—review & editing.
